# Research Into Mental Health Prediction of Community Workers Involved in the Prevention of COVID-19 Epidemic Based on Cloud Model

**DOI:** 10.3389/fpubh.2022.898148

**Published:** 2022-06-13

**Authors:** Jialing Huang

**Affiliations:** Department of Public-Based Teaching and Research, Guangxi Police College, Nanning, China

**Keywords:** psychological well-being, prediction, community staffs who preventing and controlling epidemic, cloud model, algorithm

## Abstract

To get to know the mental status of community workers involved in the prevention of COVID-19 epidemic, provide them with mental counseling and guidance, and predict their mental health status, a cloud model for the mental health prediction of community workers involved in the prevention of COVID-19 epidemic was constructed in this paper. First, the method to collect data about mental health was determined; second, the basic definition of cloud was discussed, the digital features of cloud were analyzed, and then, the cloud theory model was constructed; third, a model to predict the mental health of community workers involved in the prevention of COVID-19 epidemic was constructed based on the cloud theory, and corresponding algorithm was designed. Finally, a community was chosen as the research object to analyze and predict its mental health status. The research results suggest that the model can effectively predict the mental health status of community workers involved in the prevention of COVID-19 epidemic.

## Introduction

The COVID-19 epidemic first appeared in 2019 and quickly spread in China then. After experiencing local breakout and community-based transmission, it finally widely spread throughout China, and since April 2020, the epidemic has spread to more than 80 countries. A large number of studies suggest that acute stress disorder caused by public health emergencies such as the spread of acute infectious diseases can seriously affect people's confidence in life and work, thus doing great harm to the society. Specifically, after the outbreak of SARS in China in 2003, a large amount of patients suffered from stress disorder and mental disorder due to external trauma in many parts of China. Therefore, it is particularly urgent to alleviate such negative effects. In 2020, the World Health Organization declared the worldwide spread of COVID-pandemic, and to effectively prevent the disease, the Chinese government has adopted a lot of effective measures, including restricting the travel of people in key areas and mobilizing the whole society in the pandemic control. Also, under the leadership of governments at all levels, grassroots units have jointly formulated the epidemic prevention and control strategies. In addition, local communities, which are at the forefront of the epidemic control, have established a “four-in-one” epidemic prevention and control organ consisting of community cadres, property management workers, community policemen, and medical workers at local healthcare centers to conduct in-depth investigation into the local epidemic prevention and control (1). In the face of the epidemic, in addition to taking scientific self-protection measures, it is also necessary for us to focus on our mental health and prevent ourselves from negative emotions such as anxiety, panic, and depression.

At present, the COVID-19 epidemic has not been eliminated as sporadic cases appear one after another everywhere, posing serious threat to people's health. Thus, it is necessary to conduct medical quarantine for close contacts and suspected infected persons to prevent the further spread of the epidemic. However, as China has a large population, it is suggested that they stay at home during their quarantine period (2). Also, the frequent occurrence of new cases poses a potential risk to workplace, and the implementation of closed-off management at high-risk communities will inevitably affect the mental health of local community workers. Furthermore, community workers often face new challenges and high work intensity during the epidemic prevention, and thus, they are easy to have emotional changes. Therefore, it is necessary to timely get to know community workers' work and emotions during the epidemic prevention and analyze relevant influencing factors, thus providing reference for conducting mental intervention for them (3).

An advanced method is adopted in this paper to effectively predict the mental health of community workers involved in the prevention of COVID-19 epidemic. Conventional fuzzy sets are used as theoretical basis to effectively deal with many engineering problems, such as engineering control optimization, data analysis, efficiency prediction, and fault diagnosis. To accurately predict the mental health of community workers involved in the prevention of COVID-19 epidemic, there is a need to obtain a large amount of relevant data and analyze and evaluate the hidden information with professional expertise and skills, so as to provide strong support for subsequent epidemic prevention and control. At present, the situation is complex, but to apply cloud model to predict the mental health of community workers involved in the prevention of COVID-19 epidemic can increase the data utilization rate and reduce the investment in labor. With the concerted efforts of researchers, the theoretical basis of cloud model is more sufficient, and the model can predict the mental health of community workers involved in the prevention of COVID-19 epidemic more effectively (4).

This paper mainly focuses on the application of cloud model in the prediction of mental health of community workers involved in the prevention of COVID-19 epidemic. According to the obtained data about mental health, the multiple rule reasoning process of cloud model was established, the technical routes and methods of prediction were analyzed, and the case analysis was conducted to verify the effectiveness of the method. Based on the existing data, extracting the useful knowledge behind a large amount of data can reduce the waste of data and provide technical support for improving the accuracy of predicting mental health of community workers involved in the prevention of COVID-19 epidemic (5).

The cloud model itself combines randomness and fuzziness. It can well simulate the qualitative concept of data representation. Based on the existing data about the mental health of community workers involved in the prevention of COVID-19 epidemic, a cloud model was established to predict the mental health of community workers involved in the prevention of COVID-19 epidemic through single-rule reasoning or multi-rule reasoning. As influenced by various factors, community workers involved in the prevention of COVID-19 epidemic have random and uncertain mental health. The traditional prediction methods have difficulty in process analysis of mental health; however, the development of computer technology and Artificial Intelligent technology can provide a new method of mental health, and cloud model can well predict the mental health of community workers involved in the prevention of COVID-19 epidemic, so it has great research value. Because the cloud model has the advantage of combining randomness and fuzziness, it can improve the prediction effect of mental health of community workers involved in the prevention of COVID-19 epidemic. The proposed prediction model based on cloud model has good feasibility and effectiveness in prediction of mental health. The prediction results can be obtained without an effect of the environment and other factors.

## Collect Data About Mental Health of Community Workers Involved in The Prevention Of COVID-19 Epidemic

The specific work of COVID-19 epidemic prevention includes centralized medical observation and quarantine management, quarantined personnel inspection and management, checkpoints on duty for all people entering the city, temperature measurement in communities, disposal of daily wastes, and online mental assistance, etc. About 70% community workers participate in the inspection and management of quarantined personnel, accounting for the highest proportion. About 58.7% community workers participate in more than two of the above tasks, and some community workers even get infected due to contact with suspected COVID-19 patients. About 84.1% of community workers do not know whether there would be corresponding subsidies when accepting the pandemic control task, but they could accept the work arrangement. About 48.5% of community workers could go home every day, but due to the special pandemic control task, they have been away from home for a maximum of 35 days (6).

By scanning a QR code or clicking a link, the community epidemic control workers filled in a questionnaire about their personal information, mental health work, epidemic prevention and control work, and emotions anonymously online. Such method has good validity and reliability for the screening depressive disorders and evaluating depression severity. The questionnaire consists of 9 items designed to understand how much time the patient has been troubled by 9 problems, including depression and decreased interest, in the past 2 weeks. The score of every element is listed as follows: 0 = not at all; 1 = how many days; 2 = more than half of the days; 3 = almost every day. The total score of the scale is 27, and it was depression if the score is more than 5. The scores of 5, l0, 15, and 20 are the cutoff points for “mild,” “moderate,” “severe,” and “extreme severe” depression, respectively. The nine symptoms of depression in the questionnaire include unhappy mood, anxiety, insomnia, feebleness, anorexia, lack of self-confidence, difficulty in concentrating, irritability, and biased ideas (7).

In addition, a scale of generalized mental problems is designed in the study. The concise self-assessment anxiety scale is developed based on DSM-IV diagnostic criteria for screening for mental problems and assessing the severity of mental health conditions, which has good reliability and validity. The questionnaire consisted of 7 items, which was designed to know how much time the patient has been troubled by problems including “difficult to relax” and “too worried about all kinds of problems” in the past 2 weeks. The scores of every item are listed as follows: 0 = never, 1 = several days, 2 = more than 1 week, 3 = all time. The total score of the scale is 21, and it is anxiety if the score is more than 5. The scores of 5, 10, and 15 represent the boundary values of “mild,” “moderate,” and “severe” anxiety, respectively. The seven anxiety symptoms in the scale include nervousness, anxiety or eagerness, failure in stopping or controlling worry, worrying too much about everything, difficult to relax, unable to sit still due to anxiety, irritability, and fear of terrible things (8).

Besides, different demographic characteristics, including gender, age, marriage, education, professional title as well as occupation, and working hours, are compared in this study to determine the score of mental health of community workers involved in the prevention of COVID-19 epidemic. According to the mental health status, the respondents were divided into two groups as dependent variables, with age, marriage, education level, and working hours as independent variables. Then, unconditional logistic regression is performed to analyze the correlates of depression and anxiety. COVID-19 has brought great spiritual stimulation to the society, so mental intervention of community workers involved in the prevention of COVID-19 epidemic has become one of the important components of epidemic prevention and control. The situation has caused mental stress in epidemic prevention personnel, even acute stress disorder, and traumatic stress disorder. When acute infectious diseases are prevalent in a certain area, community workers involved in the prevention of COVID-19 epidemic may suffer from various mental problems. Due to the severity and complexity of community epidemic control, the mental health of community workers involved in the prevention of COVID-19 epidemic has been greatly impacted, and they are under tremendous work pressure and mental stress. Therefore, it is necessary to collect COVID-19 gender-specific questionnaires and gather statistics about the mental problems of community workers involved in the prevention of COVID-19 epidemic according to their gender, education level, and occupation. The most common mental emergency reactions of community workers involved in the prevention of COVID-19 epidemic include easy to get fatigue, poor sleep, and difficulty in making decisions, showing that those community workers involved in the prevention of COVID-19 epidemic have experienced health problems and are faced with huge mental problems that they need to receive appropriate mental interventions. Through the analysis of the impact of different gender, occupation, and education on the mental health of community workers involved in the prevention of COVID-19 epidemic, effective mental intervention measures can be adopted (9).

## Cloud Model Theory

### Definition and Digital Characteristics of Cloud

The quantity concept is the quantitative concept *W*, if *x*∈*W* is a stochastic implementation of concept, the certainty of *x* to *Y*, *W* → [0, 1] ,*x*∈*W*, and then, distribution of *x* on *W* is named as cloud model.μ(*x*) reflects the level of certainty that the quantitative value *x*belongs to the qualitative concept *Y*. Therefore, the cloud model can not only use the membership function μ(*x*) and describe the fuzziness of the concept, but also describe the membership function μ(*x*) and the randomness of cloud droplets (10, 11).

The digital features of cloud play an important role in cloud model. Among them, expectation *E*_*x*_ illustrates main probability of qualitative concept and is the mathematical expectation of cloud droplets in spatial distribution of universe; entropy *E*_*n*_ illustrates the uncertainty measure of qualitative concept; super entropy *H*_*e*_ illustrates the uncertainty of entropy and shows the level at which the stochastic variable corresponding to qualitative concept deviates from normal distribution.

### Cloud Model

Cloud model is an uncertainty transformation model between qualitative concept and quantitative description, which can be used to represent qualitative concept and carry out quantitative calculation. At present, the cloud model has developed from 1D to n-dimensional or even multiple dimensional, so it can be used to represent more complex natural language concepts. In this study, the forward cloud generator implementation algorithm of 1D and 2D cloud model is designed, the cloud model algorithm is implemented with MATLAB language, and the graphics of two different dimensional cloud models are drawn (12, 13).

#### 1D Forward Cloud Generator

The most basic cloud model is 1D normal cloud model, which is most commonly used in expressing qualitative concepts. The algorithm of generating cloud droplets by calculating the digital features *E*_*x*_, *E*_*n*_, and *H*_*e*_ of 1D cloud model is called 1D forward cloud generator, and vice versa.

**Algorithm 1: implementation algorithm of 1D forward cloud generator** **(****14****)**

Input: values of three numeric digital features *E*_*x*_, *E*_*n*_ and *H*_*e*_ of 1D cloud and the number of cloud droplets *i* (*i* is a positive integer).

Output: *i* 2D points (*x*_*i*_, *u*_*i*_), which are cloud droplets.

Step 1: Calculate and form a normal arbitrary number *x* with expected value of *E*_*x*_ and the typical deflection of *E*_*n*_;


(1) $$\begin{array}{l} x\;=\;Normrnd\left(E_{x},E_{n}\right) \end{array}$$

where, *Normrnd* is normal random number generating function.

Step 2: Calculate and generate a normal random number ${E_{n}}^{\prime }$ with expected value *E*_*n*_ and typical deflection *H*_*e*_;

Step 3: Calculate value of *u* by


(2) $$\begin{array}{l} u\;=\;e^{-\frac{{\left(x-E_{x}\right)}^{2}}{2{E_{n}}^{{\;}^{\prime }2}}} \end{array}$$

Step 4: Repeat *i* times of calculation steps (1) -(3), and *i*cloud drops (*x*_*i*_, *u*_*i*_) are generated.

#### 2D Forward Cloud Generator

(c) 2D cloud model is developed based on 1D model. Its numerical characteristics are six values (*E*_*x*_, *E*_*nx*_, *H*_*x*_, *E*_*y*_, *E*_*ny*_, *H*_*y*_) (15).
(d) Algorithm 2: Algorithm of 2D forward cloud implementation
(e) Input: 2D normal cloud model is represented by three numbers. Its main parameters are (*E*_*x*_, *E*_*nx*_, *H*_*x*_, *E*_*y*_, *E*_*ny*_, *H*_*y*_) and the number of cloud droplets is *n*.
(f) Output: the number of 3Dl cloud drop coordinates with number of *n*, respectively, is (*X*_*n*_, *Y*_*n*_, *U*_*n*_).

Step 1: The value of (*X*_*i*_,*Y*_*i*_) is calculated by the following expression:


(3) $$\begin{array}{l} \left(X_{i},Y_{i}\right)=Pnormrnd\left(E_{x},E_{nx},E_{y},E_{ny}\right) \end{array}$$

where *Pnormrnd* is two-dimensional random number generation function. It can be used to form 2D normal stochastic number (*X*_*i*_,*Y*_*i*_) with expected vale of (*E*_*x*_,*E*_*y*_) and standard variance of (*E*_*nx*_, *E*_*ny*_).

Step 2: The value of (*E*_*nxi*_,*E*_*nyi*_) is calculated as:


(4) $$\begin{array}{l} E_{nxi},E_{nyi}=pcg\left(E_{nx},H_{x},E_{ny},H_{y}\right) \end{array}$$

Step 3: The value of *U*_*i*_is calculated as:


(5) $$\begin{array}{l} U_{i}=e^{-\frac{1}{2}\left[\frac{{\left(X_{i}-E_{x}\right)}^{2}}{E_{nxi}^{2}}+\frac{{\left(Y_{i}-E_{y}\right)}^{2}}{E_{nyi}^{2}}\right]} \end{array}$$

Step 4: Repeat *n* times of calculation steps 1–3, and *n*cloud drops (*X*_*n*_, *Y*_*n*_, *U*_*n*_) can be formed.

## Mental Health Prediction Model for Community Workers Involved in The Prevention Of COVID-19 Epidemic Based on Cloud Theories

### Language Prediction Rules Based on Cloud Model

**Figure 1:**
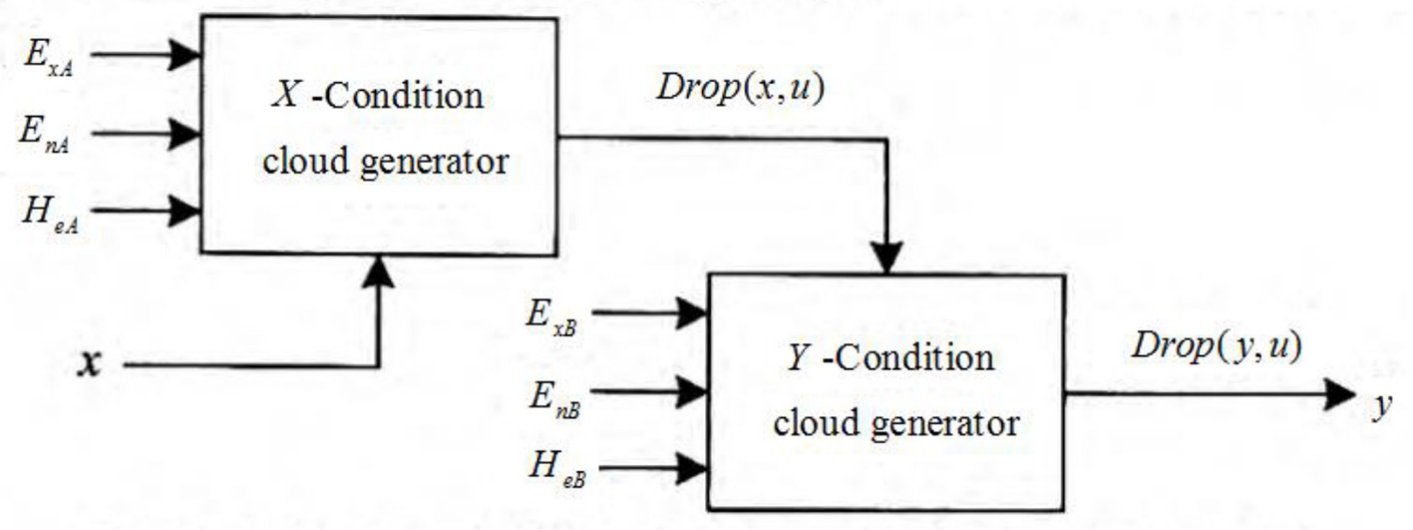
Cloud language prediction rules based on uncertain reasoning.

Cloud model can effectively realize the transition between quantitative values and corresponding qualitative concepts, represent qualitative concepts through corresponding rule generators, and realize uncertain reasoning between different concepts. Figure 1 shows the cloud language prediction rules based on the conditional cloud generator (16, 17).

In Figure 1, the connection between *X*-condition cloud generator and *Y*-condition cloud generator constitutes the corresponding cloud language prediction rules, and its corresponding algorithm is as follows (18):

Step 1: According to the qualitative concept *C*_*A*_(*E*_*xA*_, *E*_*nA*_, *H*_*eA*_) of *X*conditional cloud generator, a normally distributed random number $E_{nA}^{{\;}^{\prime }}=Norm\left(E_{nA},H_{eA}^{2}\right)$ is generated;

Step 2: Calculate the membership degree of the specific input value *x* of the *X* condition cloud generator by


(6) $$\begin{array}{l} u=e^{-\frac{{\left(x-E_{xA}\right)}^{2}}{2E_{nA}^{2}}} \end{array}$$

Step 3: According to the qualitative concept *C*_*B*_(*E*_*xB*_, *E*_*nB*_, *H*_*eB*_) of - conditional cloud generator, a normally distributed random number $E_{nB}^{{\;}^{\prime }}=Norm\left(E_{nB},H_{eB}^{2}\right)$ is generated;

Step 4: Calculate the output of a specific input value *x*. If *x*<*E*_*xA*_, the input is at the rising edge of the conditional cloud, and $b=E_{xB}-E_{nB}^{{\;}^{\prime }}\sqrt{-2\ln\;u}$; otherwise, it is at the falling edge, $b=E_{xB}+E_{nB}^{{\;}^{\prime }}\sqrt{-2\ln\;u}$.

To realize the uncertainty reasoning from qualitative concept *C*_*A*_(*E*_*xA*_, *E*_*nA*_, *H*_*eA*_) to qualitative concept *C*_*B*_(*E*_*xB*_, *E*_*nB*_, *H*_*eB*_), for the specific input value *x* in universe *U*_*A*_, the *X* conditional cloud generator randomly generates its corresponding uncertainty *u* and transfers the uncertainty of qualitative concept *A* to universe *U*_*B*_. The *Y* conditional cloud generator generates and outputs a random cloud drop *drop*(*b, u*) under the control of uncertainty *u*, so the output value *b* of *Y*conditional cloud also has uncertainty. In other words, the transmission of uncertainty is realized in cloud reasoning.

### Cloud Model Representation of Concepts Related to Mental Health Prediction of Community Workers Involved in The Prevention of COVID-19 Epidemic

#### Community Workers Involved in the Prevention of COVID-19 Epidemic

To predict the failure of power transmission and transformation equipment, it is necessary to input the historical data of power transmission and transformation equipment and build a cloud reflecting the two related qualitative concepts of the mental health and the possibility of mental obstacles of community workers involved in the prevention of COVID-19 epidemic using the reverse cloud generator (19). If the input data are more than 10 groups, the expected value obtained has high accuracy and small error. Therefore, the mental health data of 10 groups of community workers involved in the prevention of COVID-19 epidemic were used in this study.

Set *X* as statistical mean of mental health index of community workers involved in the prevention of COVID-19 epidemic in the year related to maximum equipment failure ratio, and *u*(*x*) as the level at which a certain mental health index *x*_*i*_ of equipment belongs to mean value *X* of mental health index in the community with most serious mental disorders. Therefore, the membership computing means of mental health parameter of the community workers involved in the prevention of COVID-19 epidemic are (20).


(7) $$\begin{array}{l} u\left(x\right)=\{\begin{array}{c} \frac{x_{i}}{X},\text{when }x_{i}<X\\ 1,\text{when }x_{i}\geq X \end{array} \end{array}$$

To form the trend cloud of mental health status of community workers involved in the prevention of COVID-19 epidemic in the corresponding state, it is assumed that *p*_*i*_(*i* = 1, 2, ⋯ , 10) is the mental health level of 10 groups of community workers involved in the prevention of COVID-19 epidemic. *P* is the level with the most serious mental problems of epidemic prevention workers in 10 communities, *u*(*p*) is the mental health level *p*_*i*_ of pandemic control workers in one community, which belongs to the degree of the maximum mental health level *P* of pandemic control workers in 10 groups of data. Then, the following expression can be obtained:


(8) $$\begin{array}{l} u\left(p\right)=\{\begin{array}{c} \frac{p_{i}}{P},\text{when }p_{i}<P\\ 1,\text{when }p_{i}\geq P \end{array} \end{array}$$

After reverse cloud generator is applied, historical data and appropriate membership mentioned above are inputted to form numeric features of 3D cloud models, respectively, and status-related cloud *C*_*A*_(*E*_*xA*_, *E*_*nA*_, *H*_*eA*_) illustrating the mental health status of corresponding power transmission and transformation equipment and the fault trend cloud *C*_*B*_(*E*_*xB*_, *E*_*nB*_, *H*_*eB*_) illustrating possibility of fault occurrence have got.

The algorithm of the reverse cloud generator is as follows:

Step 1: Based on normal cloud expectation curve expression $y=e-\frac{{\left(x-E_{x}\right)}^{2}}{2E_{n}^{2}}$, known cloud droplets can be used to get $E_{x}^{\wedge }$ by fitting operation;

Step 2: Eliminate points with *y*>0.999, leaving *m* cloud drops;

Step 3: Calculate $E_{n}^{\wedge }$ by the following expression:


(9) $$\begin{array}{l} \begin{array}{l} {E^{\prime }}_{n}=\frac{\left|x-E_{x}^{\wedge }\right|}{\sqrt{-2\ln\;y}} \end{array} \end{array}$$

Step 4: Calculate $H_{e}^{\wedge }$ by the following expression:


(10) $$\begin{array}{l} \begin{array}{l} H_{e}^{\wedge }=\frac{\sqrt{\overset{m}{\underset{i=1}{\sum^{\text{​}}}}{\left({E^{\prime }}_{ni}^{\;}-E_{n}^{\wedge }\right)}^{2}}}{\sqrt{m-1}} \end{array} \end{array}$$

Take the state association cloud *C*_*A*_(*E*_*xA*_, *E*_*nA*_, *H*_*eA*_) and fault trend cloud *C*_*B*_(*E*_*xB*_, *E*_*nB*_, *H*_*eB*_) as the qualitative concept cloud and cloud reasoning rules corresponding to *X* condition cloud and *Y* condition cloud, so as to realize the reasoning and prediction of mental health status of community workers involved in the prevention of COVID-19 epidemic.

Step 1: Collect historical reliability data and state prediction data;

Step 2: Define the calculation method of conditional cloud membership to form input cloud droplets;

Step 3: Based on the reverse cloud generator, the state correlation cloud and the current trend cloud of mental disorders of community workers involved in the prevention of COVID-19 epidemic are generated;

Step 4: Conditional cloud interconnection constitutes a cloud prediction reasoning model;

Step 5: Calculate the mental health level of community workers involved in the prevention of COVID-19 epidemic under the current state, and judge whether it is >0, if the mental health level is larger than 0, the predicted mental health level is output, otherwise, return to Step 4.

## Mental Health Prediction Simulation For Community Workers Involved in The Prevention of COVID-19 Epidemic

Community workers involved in the prevention of COVID-19 epidemic have various mental problems. In this research, the mental health level of community workers involved in the prevention of COVID-19 epidemic is predicted at all levels. *w* is defined as the mental health level of community workers involved in the prevention of COVID-19 epidemic and *s*(*w*)as the validity function of mental health level of community workers involved in the prevention of COVID-19 epidemic, i.e., the severity of mental problems.

The preference utility function meets the requirements of *s*′(*w*)>0 and *s*″(*w*)>0, that is, the degree of mental disorder increases and the severity of mental problems increases faster, which is in line with the actual situation of mental health of community workers involved in the prevention of COVID-19 epidemic. Therefore, this paper uses the preference utility function to represent the fault severity (γ = 1).


(11) $$\begin{array}{l} S\left(w\right)\;=\;0.576\left(e^{w}-1\right) \end{array}$$

The mental health level is defined as the product of the probability of mental disorder and the consequences of mental problem, which is expressed by


(12) $$\begin{array}{l} R=0.576P\cdot \left(e^{w}-1\right) \end{array}$$

where *R* denotes the mental well-being level value, *P* denotes the probability of mental problem.

**Figure 2:**
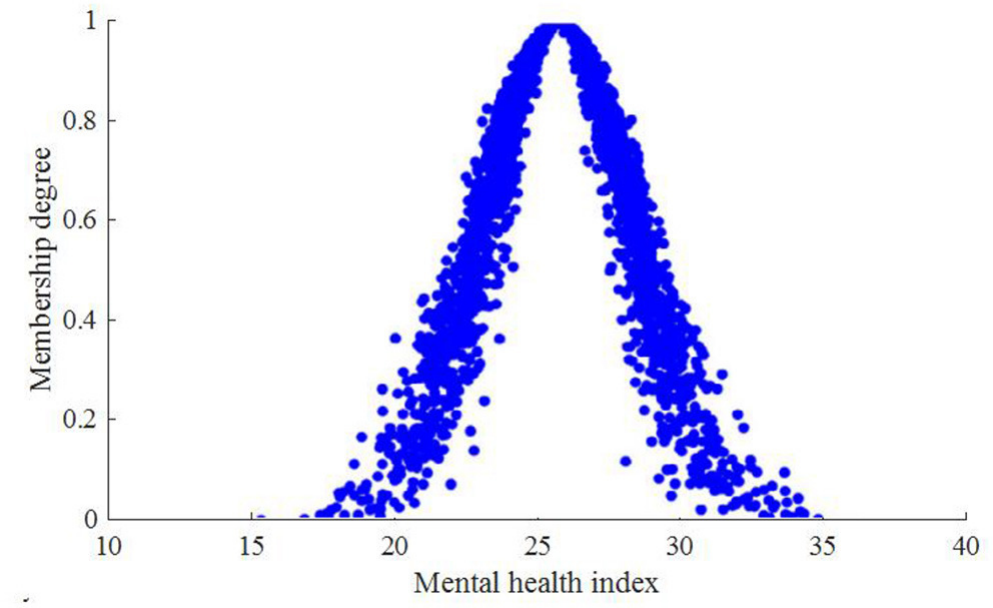
Distribution of mental health indexes of community workers involved in the prevention of COVID-19 epidemic.

**Figure 3:**
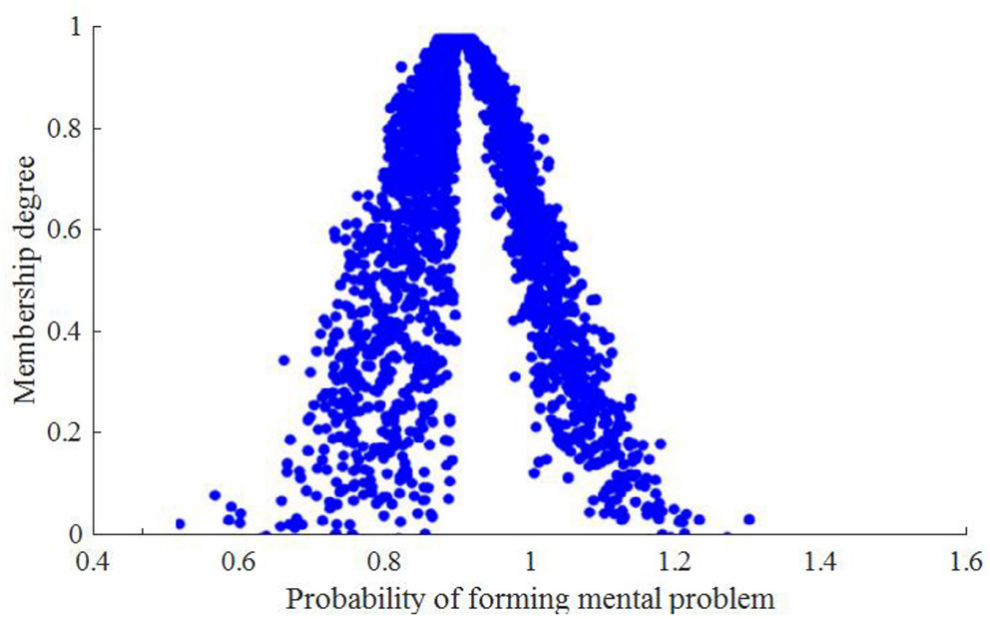
Probability of forming mental problem for community workers involved in the prevention of COVID-19 epidemic.

A community is selected to as the research object. The mental problem of community workers involved in the prevention of COVID-19 epidemic mainly concludes force, interpersonal sensitivity, depression, anxiety, hostile, phobic paranoid, psychotic, and sleeping state. According to the above method, the mental health index status correlation cloud and current mental problem trend cloud of community workers involved in the prevention of COVID-19 epidemic are *C*_*A*_(22.4, 5.043, 0.325) and *C*_*B*_(0.325, 0.325, 0.032), respectively. Since a cloud drop in cloud model is only the quantitative implement of related qualitative concept, the more cloud drops are, the more they can show whole features of corresponding qualitative concept. Here, 600 predicted cloud drops are formed based on numeric features of the cloud stochastically, and corresponding cloud dispersion distribution is obtained, as shown in Figures 2, 3.

The analysis results show that expected value of mental health index of community workers involved in the prevention of COVID-19 epidemic is related to the expected value of mental disorders. When the mental health index is 25.6, the corresponding probability of forming mental problem is 0.93.

The probability of forming mental problems and mental health index is as shown in Table 1.

**Table 1:** Probability of psychological well-being problem and psychological well-being index.

**Mental problem**	**Probability of mental problem**	**Mental health index**
Force	0.24	32
Interpersonal sensitivity	0.32	25
Depression	0.43	27
Anxiety	0.37	26
Hostile	0.42	36
Phobic paranoid	0.26	18
Psychotic	0.22	22
Sleeping state	0.29	34

The calculation results show that the prediction results of mental health of community workers involved in the prevention of COVID-19 epidemic are in line with the statistical law of mental problems of community workers involved in the prevention of COVID-19 epidemic, and the prediction error is less than 5%. The prediction of mental health level in this study is mainly applied to the mental intervention of community workers involved in the prevention of COVID-19 epidemic, and the accuracy meets the requirements. The traditional method is used to predict mental health of the same samples, and the prediction error is greater than 5%; therefore, the proposed in this paper is better than traditional methods.

## Conclusions

In this study, a method to predict the mental health level of community workers involved in the prevention of COVID-19 epidemic is proposed based on cloud prediction model. Mental problems of community workers involved in the prevention of COVID-19 epidemic are caused by many uncertain factors, which are random and fuzzy. In this study, a cloud model is applied to the uncertain reasoning prediction of mental problems of community workers involved in the prevention of COVID-19 epidemic, and the cloud language prediction rules based on conditional cloud generator are constructed. A model to predict the mental health level of community workers involved in the prevention of COVID-19 epidemic is established based on cloud reasoning. According to the utility theory, a set of prediction index system of mental health level is proposed, including compulsion, interpersonal sensitivity, expression, anxiety, hostile, phobic paranoia, psychosis, and sleeping state. The mental problem prediction method proposed in this paper can scientifically and reasonably predict the mental health level of community workers involved in the prevention of COVID-19 epidemic and comprehensively and objectively analyze the mental problems of community workers involved in the prevention of COVID-19 epidemic. Therefore, the method can well promote the smooth progress of COVID-19 epidemic prevention and control at the community level.

## Data Availability Statement

The original contributions presented in the study are included in the article/supplementary material, further inquiries can be directed to the corresponding author.

## Author Contributions

JH is in charging of writing and data analysis.

## Conflict of Interest

The author declares that the research was conducted in the absence of any commercial or financial relationships that could be construed as a potential conflict of interest.

## Publisher's Note

All claims expressed in this article are solely those of the authors and do not necessarily represent those of their affiliated organizations, or those of the publisher, the editors and the reviewers. Any product that may be evaluated in this article, or claim that may be made by its manufacturer, is not guaranteed or endorsed by the publisher.
